# An Eddy Current Testing Platform System for Pipe Defect Inspection Based on an Optimized Eddy Current Technique Probe Design

**DOI:** 10.3390/s17030579

**Published:** 2017-03-13

**Authors:** Damhuji Rifai, Ahmed N. Abdalla, Ramdan Razali, Kharudin Ali, Moneer A. Faraj

**Affiliations:** 1 Faculty of Engineering Technology, Universiti Malaysia Pahang, Gambang, Pahang 26300, Malaysia; damhuji@tatiuc.edu.my (D.R.); ramdan@ump.edu.my (R.R.); mod84_91@yahoo.com (M.A.F.); 2 Faculty of Electrical & Automation Engineering Technology, TATI University College, Kemaman 26000, Malaysia; kharudin@tatiuc.edu.my

**Keywords:** non-destructive testing, GMR, pipeline inspection

## Abstract

The use of the eddy current technique (ECT) for the non-destructive testing of conducting materials has become increasingly important in the past few years. The use of the non-destructive ECT plays a key role in the ensuring the safety and integrity of the large industrial structures such as oil and gas pipelines. This paper introduce a novel ECT probe design integrated with the distributed ECT inspection system (DSECT) use for crack inspection on inner ferromagnetic pipes. The system consists of an array of giant magneto-resistive (GMR) sensors, a pneumatic system, a rotating magnetic field excitation source and a host PC acting as the data analysis center. Probe design parameters, namely probe diameter, an excitation coil and the number of GMR sensors in the array sensor is optimized using numerical optimization based on the desirability approach. The main benefits of DSECT can be seen in terms of its modularity and flexibility for the use of different types of magnetic transducers/sensors, and signals of a different nature with either digital or analog outputs, making it suited for the ECT probe design using an array of GMR magnetic sensors. A real-time application of the DSECT distributed system for ECT inspection can be exploited for the inspection of 70 mm carbon steel pipe. In order to predict the axial and circumference defect detection, a mathematical model is developed based on the technique known as response surface methodology (RSM). The inspection results of a carbon steel pipe sample with artificial defects indicate that the system design is highly efficient.

## 1. Introduction

Pipelines are used in virtually every nation around the globe to transport oil and gas from the fields to the market. While pipes are cheaper than other means of transportation, this cost saving alternative comes with a major price: pipes are subject to cracks and corrosion which in turn can cause leakage and environmental damage. Oil spills, gas leaks, and their associated environmental problems have become a serious and major concern in the oil and gas industry, and consequently, this has led to significant losses in revenue, severe disruption of operations as well as a persistent threat to marine life and the ecosystem. This accidental discharge of petroleum products on/offshore has hitherto caused untold and unimaginable environmental hazards and economic loss that requires urgent remedial action and attention. The cases that were investigated found faults in the pipeline degradation system. Zou et al. [1] found that the degradation of mechanisms that have been identified fall into the categories of mechanical damage, corrosion, environmental cracking, and original manufacturing defects. Therefore, the assessment of the integrity of a pipeline can be accomplished by assessing the defects that occur on the pipe.

To ascertain the trustworthiness and cost-effectiveness of a structure, an effectual technology for sensing and assessing the pipeline structure that can be used is the non-destructive testing (NDT). The destructive and non-destructive testing (NDT) are required to ensure the reliability of a pipeline prior to it being operated. In addition, swift assessments of any flaws in the structures are very important to alleviate the workload of the testers. There are various inspection methods to ensure the reliability of pipelines. The type of inspection method selected depends largely on the type of defect, the type of material, whether the pipeline is accessible or otherwise, as well as the financial considerations.

Eddy current testing methods have been developed as the main technique for pipe inspection. They can also be utilised to identify interior corrosion resulting from metal deficits. The eddy current inspection has various features that make it an appropriate option for pipe inspection purposes. Most importantly eddy current sensors do not require any mechanical contact between the ECT probe and the test object [2]. In addition, by amending the frequency of the excitation current, the inspection depth can be adjusted. The method is highly sensitivity to defects in the interior of the pipe. Traditional ECT methods detect variations in the magnetic field using coils as sensors [3]. The coil sensors are fundamentally limited by their poor sensitivity at low frequencies. Regrettably, the examination of thick samples and subsurface defects require sensitivity at low frequency [4]. This limitation suggests that conventional eddy current testing probes are reaching their development limits and that new sensors are needed to push back the present boundaries of flaw detection. The superiority of magnetometer (MR) sensors over coil systems has been established as a magnetic field detection method in ECT probes. Optimum information from the component being tested can be achieved by employing highly sensitivity magnetic detectors and not conventional coil detectors. Du et al. [5] proposed a magnetic sensor to monitor outer or inner pipe wall defects. The experimental results confirmed that the application of GMR sensors was able to measure the defective magnetic profile in the ferromagnetic pipe with precision. In addition, the study indicates the defects of pipe inspection using ECT are dependent on various variables including flaws, sensor lift-off, geometry and the properties of the material. In another study, Le et al. [6] proposed integrating a 71 GMR sensor for the purpose of inspecting the corrosion in a small bore piping system. The experiments showed the implementation of a huge number of arrays in the GMR sensors could improve the evaluation of cracks in pipe inspection for defect parameters, for example length, the volume of cracks as well as their depth. Cheng [7] performed experimental investigations on the implementation of the Pulsed Eddy Current Testing and GMR sensor in measuring the problem of wall-thinning of carbon steel pipes. The initial results suggested that the ECT system constructed using the GMR sensors was capable of measuring a magnetic field signal at a micro-Gauss level. This superior ability made the system suitable for small defect inspection in inner pipes. Vacher et al. [8] proposed a high-resolution ECT probe using an array of GMR sensors for the inspection of a 316 L stainless pipe to measure even a small defect of less than 100 μm.

According to the state of the art, the probability of detection for eddy current techniques can be improved by optimizing the probe design. In recent years, several studies have focused on optimization of the ECT probe design for defect detection. Rocha et al. [9] proposed an ECT probe design based on velocity induced eddy currents to detect surface defects. Commercial simulation software was used for the optimization and design of the probe. Their experimental results confirmed that the proposed probe design was able to detect defects in the conductive materials where motion is involved. A biorthogonal rectangular probe was developed by Zhou et al. [10]. Simulation results showed the new biorthogonal rectangular probe has a lesser effect on the lift-off with higher sensitivity detection. Meanwhile, Cardoso et al. [11] optimized the sensor configuration in the eddy current probe design. A finite element modelling simulation was used to measure the accuracy detection of the probe design. Comparison of experimental and simulation date proved the accuracy of the proposed probe. Research by Rosado et al. [12] reported the influence of the geometrical parameters of an eddy currents planar probe in eddy current testing. The findings showed that modifications of the studied parameters can substantially improve the probe performance. In a different study, Li and Lowther [13] proposed a methodology for determining the optimal sensor parameters for ECT probe design. Simulation results showed the proposed sensor geometry improved the probe sensitivity to depth changes in a crack.

The previous literature reveals that the level of sensitivity of the ECT probe can be addressed through optimization of the probe design parameters and the use of magnetic sensors in place of the conventional pickup coils [14]. Although there is abundant research about the implementation of GMR sensor and optimize probe design in a non-destructive ECT, only a small amount of it has focused on the optimization of the ECT probe design for pipe inspections. Thus, an in depth study of the probe design for ECT pipe inspections is crucial. This research aims to illustrate the development of an ECT system based on an optimized ECT probe design. RSM was utilized to enhance the design parameters of the probe to enable an optimum detection response of axial and circumference defects.

## 2. Architecture of the Distributed System for Eddy Current Testing (DSECT) Inspection

**Figure 1:**
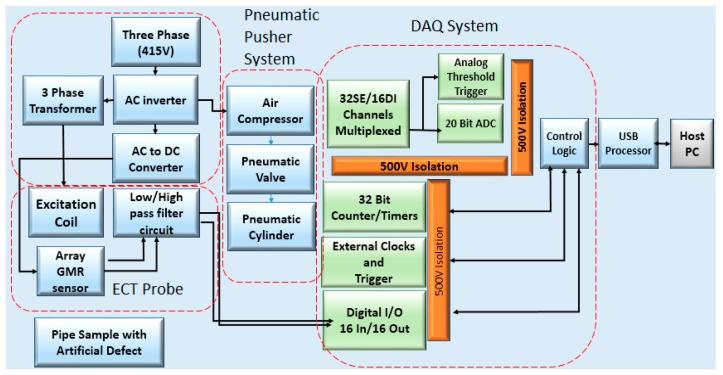
Architecture of Distributed System for Eddy Current Testing (DSECT).

A Distributed System for Eddy Current Testing (DSECT) was developed for automatic pipe defect inspection. For development planning and enabling easy troubleshooting, the system is divided into four modules, namely probe design, power system, electropneumatic system and data acquisition. The DSECT system architecture is shown in Figure 1 below.

The power for this system is sourced from a three-phase 415 V supply which then controls the supply channel for the respective components in this system. A variable frequency drive is used to control the supply frequency. Multi-frequency power is required for the ECT excitation coil for defect detection at different depths. Overall, this system requires three different supplies: DC 5 V and 24 V for the purpose of supplying the sensor array and pneumatic system. 240 V AC supply is required to enable the stable functioning of the air compressor. To generate the rotating magnetic field, a 3-phase supply with amplitude of 16 VAC was obtained by stepping down the voltage from the main source. For safety reasons, the installation of the supply for the system is carefully handled.

**Figure 2:**
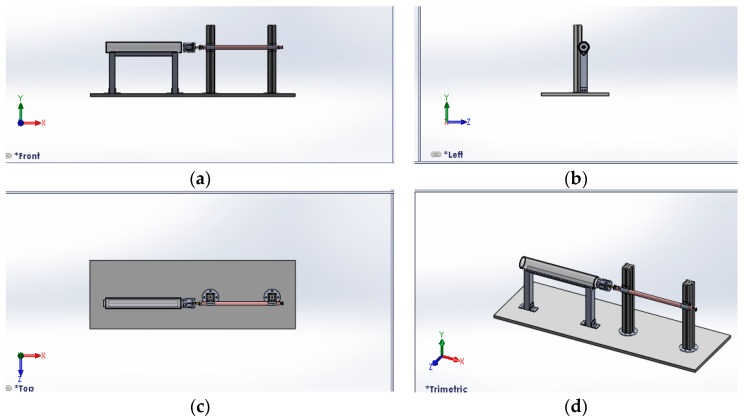
Design of the Distributed System for Eddy Current Testing (DSECT) (**a**) Front view (**b**) Side view (**c**) Top view (**d**) Angle view.

The DSECT system illustrated in Figure 2 is intended for high precision defect inspection in carbon steel pipe and serves as the study platform for the Eddy Current Testing Signal analysis in terms of defect detection and characterization. A previous study of ECT non-destructive testing provided strong evidence that the probe design, the configuration of the GMR sensor array and the frequencies of the magnetic excitation are the main factors to ensure accurate defect inspection. For pipe inspection, the cylinder structure of a pipe will cause different positions of each GMR sensor in the array sensor. Hence, an optimum number of sensors are needed to scan the entire inner pipe surface. For optimum defect detection during the pipe inspection, a response surface methodology method is utilised to find the optimal design parameter for the DSECT probe.

The functional diagram of the GMR sensor array, as illustrated in Figure 1, is constructed on the basis of the Arduino low power microcontrollers. Different types of sensors/transducers can be connected with the digital serialized frame output or analog output the by employing the Arduino microcontroller. This microcontroller allows the system to be flexible enough to be used with different types of sensors/transducers. The key function of this microcontroller is to filter unwanted signals. In other words, very low signal strength means that the measurements will incur a considerable amount of errors. A weak GMR sensor signal can be caused by various reasons such as exciting coil frequency and skin depth effect, material magnetic permeability, probe lift-off and pipe material conductivity.

The system runs using selectable sampling frequencies of 1MHz (as the default sampling frequency) as well as employing both the synchronous or asynchronous data acquisition modes. In the case of the synchronous operation mode, an external 2-wire links the GMR sensor array. This permits the synchronization feature to be more flexible and to run faster.

### 2.1. Data Acquisition System

In order to accomplish the purpose of real-time defect inspection in ferromagnetic pipes, the DSECT system was designed base on a DT 9844 data acquisition board from Data Translation (Data Translation, Norton, Cupertino, CA, USA). The DT9844 is built to be highly accurate with 1 MHz sampling rate and it has a 20-bit resolution USB data acquisition module which offers incomparable efficiency in terms of its operation. In this module, the board has distinctive circuitry to lessen any possible crosstalk between channels. The ±500 V tri-sectional galvanic isolation allows the board to able to avoid any digital interference. This provides accurate analog signals that in turn ensure signal reliability. It is found that even for the most sophisticated applications, a high-performance measurement solution can be provided when the system is merged with the QuickDAQ software (Data Translation, Norton, Cupertino, CA, USA).

**Figure 3:**
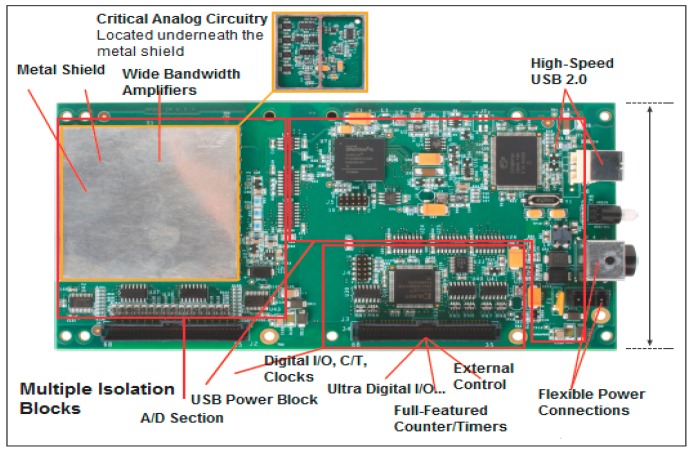
High-speed DAQ card (DT 9844) for the DSECT system.

In Figure 3, the DAQ modules are connected to the host PC by USB 2.0 channel for each GMR sensor. A USB 2.0 protocol was selected to link the host PC and the DAQ module, using 1 MSamples/second to cater to a huge number of GMR sensors and the considerable amount of data transmitted. Theoretically, it accepts up to 32 high-speed modules in terms of grouped connection and linked to a USB 2.0 with an external power source.

### 2.2. Pneumatic Pusher System

**Figure 4:**
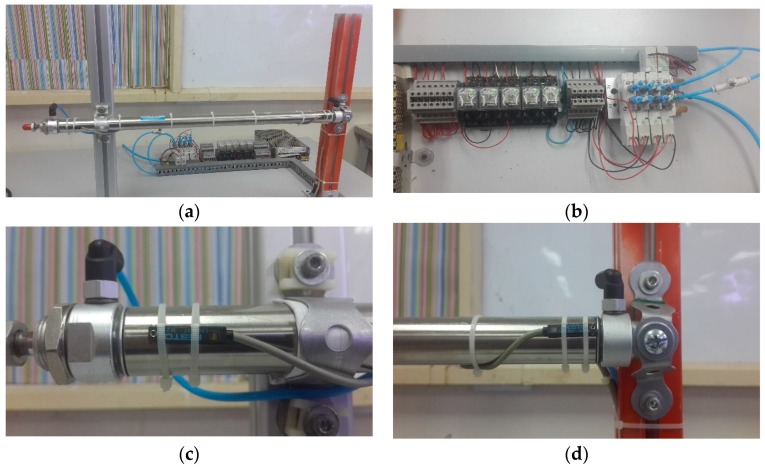
DSECT Pusher system (**a**) Hardware setup (**b**) Pneumatic system (**c**) Festo magnetic reed sensor at the end of cylinder (**d**) Festo magnetic reed sensor at the beginning of cylinder.

Figure 4a shows the entire pusher system for the DSECT system, including a pneumatic circuit and PLC controller system. The H-shape structured system is supported by two legs that can expand the eddy current probe. It can be operated via the control of a pneumatic cylinder, as illustrated in Figure 4b. The pneumatic cylinder can extend 70 mm. Thus, the distance coverage scanning of the eddy current probe for the inner pipe inspection is 50 mm. A FESTO magnetic reed sensor is attached at both ends of the pneumatic cylinder to detect the movement of the pneumatic rod cylinder as shown in Figure 4c,d. Both magnetic sensors ensure the movement of the cylinder rod is fully extended and retracted. The output sensor is read by a PLC controller as an input for measuring the speed of inspection using this system. The speed of scanning probe is controlled by using an exhaust air flow control valve which is programmed in the PLC controller to push the ECT probe with a speed of up to 0.8 m/s. The simulation of this pneumatic pusher system is done using the FluidSIM Pneumatic software (FESTO, Stuttgart, Germany). For the purpose of investigating the efficiency of the probe design in scanning the pipe defect, the system is programmed to be able to operate at different speeds.

### 2.3. Response Surface Methodology (RSM)

Response surface methodology (RSM) was first introduced by Box and Draper in 1987 [15]. This methodology was used for deriving second order empirical models from experimental data sets and subsequently used for optimization of the response surface that is influenced by various process parameters. This statistical empirical model is only an approximation to reality, nevertheless, RSM has been used effectively in various applications for optimizing products and services. According to Box and Draper, if all variable process parameters are assumed to be measurable, the response surface can be expressed as follows:

(1) $$Y=f\left(X_{1u},X_{2u},\ldots ..,X_{iu}\right)+E_{u}$$

The first goal for RSM is to find the optimum response. The second goal is how the response changes in a given direction by adjusting the design variables. It is required to find a suitable approximation defining true functional relationship between the independent variables and response variable. Usually, a second order model is developed in response surface methodology:

(2) $$y_{k}=a_{0}+\overset{3}{\underset{i=0}{\sum }}a_{i}x_{i}+\overset{3}{\underset{i=1}{\sum }}\overset{3}{\underset{j=i}{\sum }}a_{\mathrm{ij}}x_{i}x_{j}$$

where *y_k_* (*k* = 1, 2, 3 and 4) are the input variables influencing the response. *x_i_* (*i* = 1, 2 and 3) are the input variables and *a*_0_, *a_i_* (*i* = 1, 2, 3) and *a_ij_* (*i* = 1, 2, 3; *j* = *i*, ..., 3) are unknown coefficients.

## 3. Design and Operational Principles of the DSECT Probe

The proposed ECT probe for this DSECT system operates using the send-receive mode with excitation and the GMR array detection sensor. In order to detect all the orientations of defects, it is effective to generate the eddy current flow in the cross direction with respect to the defect. In practice, the presence of different types of defects make it is almost impossible to create the net current flow in the axial direction for different types of defect. Hence, it is suggested that a rotating magnetic source be used as a magnetic excitation.

**Figure 5:**
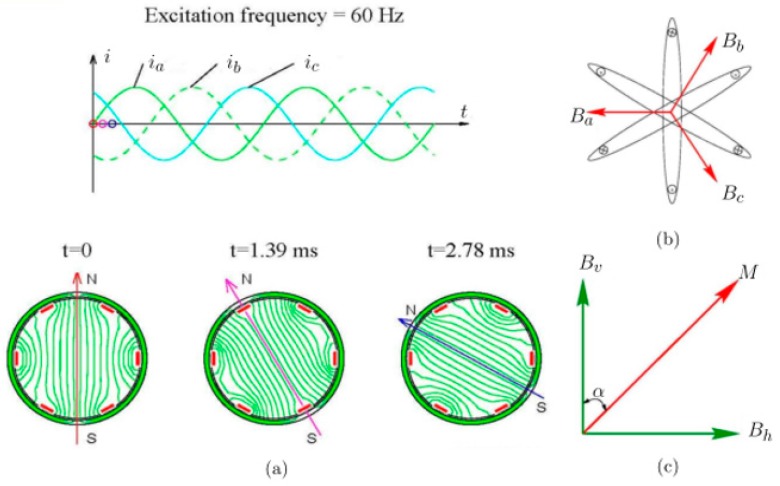
Principle of the rotating field: (**a**) three-phase currents (**b**) flux due to currents in three-phase winding (**c**) horizontal and vertical components of the resultant magnetic flux.

A three-phase AC power source is used to drive the excitation coil in order to generate a rotating magnetic field. In principle, the rotating magnetic field generated by the excitation coil probe will be sensitive to all types of defect, whether axial or circumferential. This is because the rotating magnetic field flux will be crossed over and intersected with all kinds of defects in the cylinder pipe. The rotating field principle is a fundamental property of all electrical machines and consists of three coils that are identical, located on axes that are 120 degrees physically apart and supplied from a balanced three-phase supply (i.e., the difference between the phases of exactly 120 degrees). An illustration of the magnetic field generated by each identical coil is shown in Figure 5.

The currents through these windings are represented as:

(3) $$i_{a}=\sqrt{2}i\cos\omega t$$

(4) $$i_{b}=\sqrt{2}i\cos\left(\omega t-\frac{2}{3}\pi \right)$$

(5) $$i_{c}=\sqrt{2}i\cos\left(\omega t-\frac{4}{3}\pi \right)$$

The magneto-motive force (MMF) due to these three windings can be calculated by the following equations:

(6) $$f_{a}\left(t,\theta \right)=F_{\emptyset }\cos\theta \;\omega t=\frac{1}{2}F_{\emptyset }\cos\left(\omega t+\theta \right)+\frac{1}{2}F_{\emptyset }\cos\left(\omega t-\theta \right)$$

(7) $$f_{b}\left(t,\theta \right)=F_{\emptyset }\cos\left(\theta -\frac{2}{3}\pi \right)\cos\left(\omega t-\frac{2}{3}\pi \right)\;\;=\frac{1}{2}F_{\emptyset }\cos\left(\omega t-\theta \right)+\frac{1}{2}F_{\emptyset }\cos\left(\omega t+\theta -\frac{4}{3}\pi \right)$$

(8) $$f_{c}\left(t,\theta \right)=F_{\emptyset }\cos\left(\theta -\frac{4}{3}\pi \right)\cos\left(\omega t-\frac{4}{3}\pi \right)\;\;=\frac{1}{2}F_{\emptyset }\cos\left(\omega t-\theta \right)+\frac{1}{2}F_{\emptyset }\cos\left(\omega t+\theta -\frac{2}{3}\pi \right)$$

(9) $$f=f_{a}+f_{b}+f_{c}=\frac{3}{2}F_{\emptyset }\cos\left(\omega t-\theta \right)$$

The vector summation of these MMFs has constant amplitude and rotates at the same angular frequency ω, which is similar as a magnetic dipole. It rotates circumferentially at a rate dictated by the excitation frequency.

**Figure 6:**
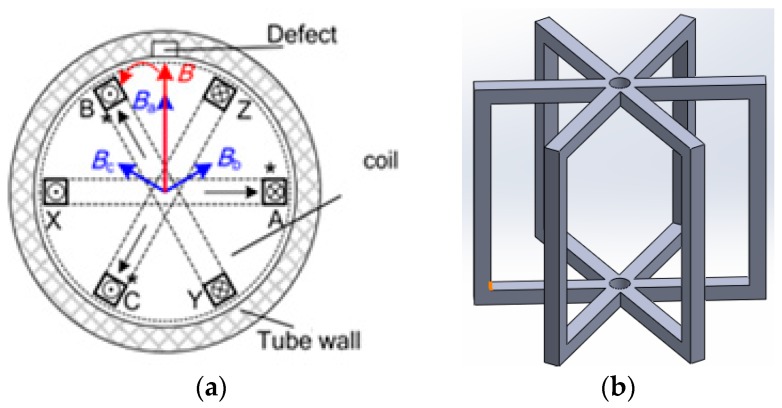
Rotating field windings and bobbin pickup coil: (**a**) Three phase excitation windings; (**b**) 3D model of three coils winding for probe excitation.

The principle can also be explained from the point of view of the magnetic flux density. Figure 6a shows the magnetic flux density components B_a_, B_b_, and B_c_ associated with three windings AX, BY, and CZ that are perpendicular to the plane of each winding. The three components synthesize a total magnetic flux density vector B with constant amplitude at any given time, which rotates at a similar rate as the excitation source. The magnetic field in the cross-section of the pipe mainly rotates in the radial direction which flows circularly about the radial axis, which makes the probe sensitive to cracks of all orientations. If there is no defect in the pipe wall, the magnetic flux is mainly in a radial direction, and there is no axial magnetic flux on the cross-section. However, if a defect is present close to the center plane, causing a variation in the radial magnetic fields, an axial magnetic field component occurs. Therefore, the defect is detectable by a probe with an array of giant magnetoresistance (GMR) sensors oriented to measure the axial component.

**Figure 7:**
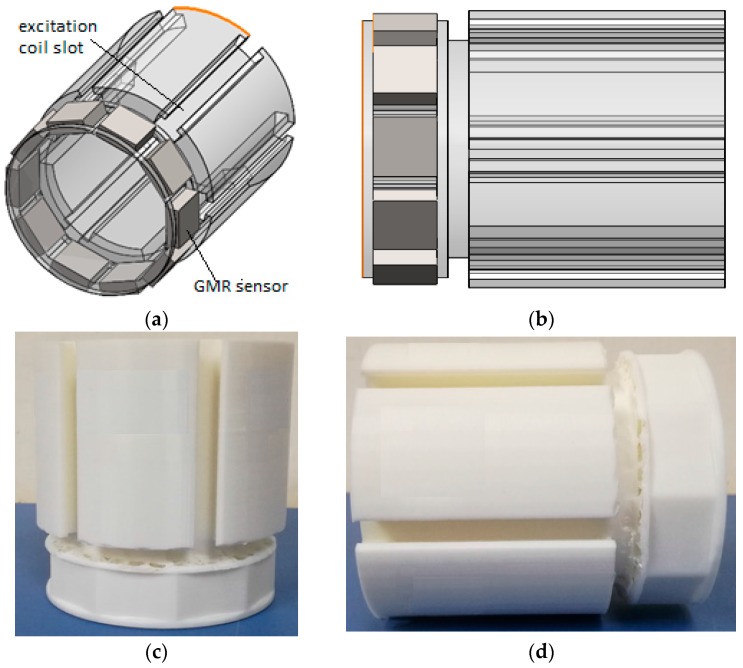
Proposed ECT probe design for DSECT system: (**a**) trimetric view (**b**) left view (**c**,**d**) probe prototype.

The centre of the proposed ECT probe consists of an array of GMR sensors located on the outside of the cylindrical probe. The probe geometry is acquired in order to ensure that the system is reliable for a non-destructive evaluation (NDE) of a defect in the inner carbon steel pipe. It can provide an effective solution to detect cracks in specimens of different pipe materials. The proposed ECT probe design is shown in Figure 7.

The DSECT probe’s dimensions have been fixed for the inspection of pipe with a diameter of 70 mm. The proposed probe design has dimensions of 60 mm diameter and a total height of 80 mm. This probe has a simple structure and does not require a motor to rotate the magnetic field, so it allows pipe inspections at a high speed.

### 3.1. Rotating Magnetic Field

**Figure 8:**
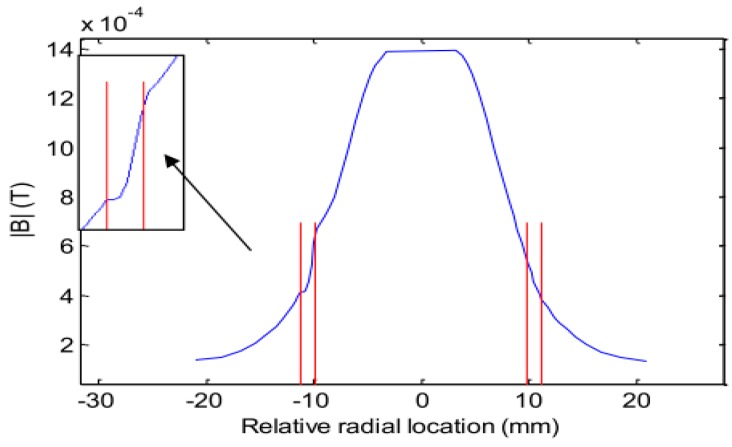
Magnetic flux density decay along diameter direction.

The magnetic flux density of the rotating field is uniformly distributed in the center plane of the windings (near the axis of the pipe), and decays exponentially in the radial direction, as shown in Figure 8, within the double red lines representing the pipe wall. The figure presents the magnetic flux density amplitude distribution along the radial direction on the plane of the pickup sensor. The presence of a through wall square hole (3.5 × 4 mm) in the pipe wall is shown in the zoomed-in area of the magnetic field on the left-hand side of the curve.

**Figure 9:**
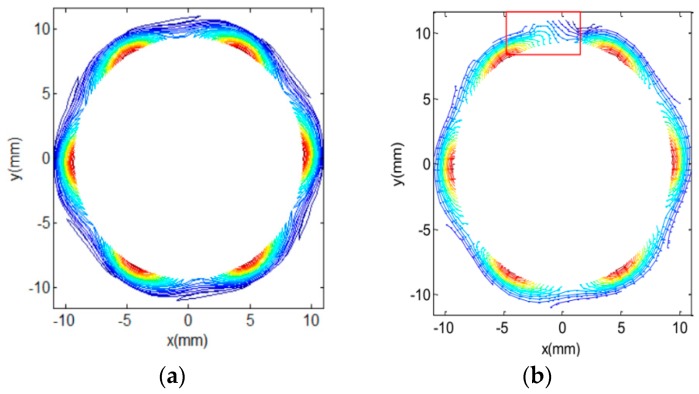
Amplitude contour of magnetic field component on the xy plane: (**a**) defect-free; (**b**) defect at 90°.

If there is no flaw in the wall of the pipe, the total magnetic field at the center of the coil is purely radial, with no vertical component. The vertical component arises from the eddy current perturbations caused by the flaws in the pipe wall. In this manner, the probe is automatically self-nulling. The rotating magnetic field vector is sinusoidal in time as well as space along the circumference of the pipe wall. Figure 9a shows the magnetic flux density contour around the pipe wall at an instant of time for the case which has no defect. Figure 9b shows the field contour in the presence of a through-wall hole. The flaws disturbs the induced eddy current and causes a variation in the magnetic flux density around defect area in the pipe wall.

### 3.2. GMR Array Sensor

The thin-film constructions comprised of non-magnetic conductive layers and alternating ferromagnetic layers detected in the quantum mechanical magnetoresistance effect are known as giant magnetoresistance (GMR) devices. Rifai [4] asserted that a GMR sensor has two or more ferromagnetic thin films separated by thin non-magnetic conducting layers. The resistance can be cut down by up to 20% compared to that of a conventional anisotropic magnetoresistive (AMR) materials when the GMR sensor is exposed to a magnetic field. One of the main functions of the GMR is magnetic field sensing. GMR sensors have low cost and low power consumption with small dimensions compared with Hall sensors. An advantage of GMR sensors is that they have high sensitivity over an extensive range of frequencies from the DC to MHz frequencies. However, GMR sensors need to be inclined properly for bipolar operation. This can be achieved easily with the electromagnetic field of the DC wire or the magnetic field of permanent magnets. The GMR magnetometer used to fabricate the array of GMR sensors in our work is a *AA002* from Nonvolatile Electronics (NVE corporation, Minneapolis, MN, USA).

**Figure 10:**
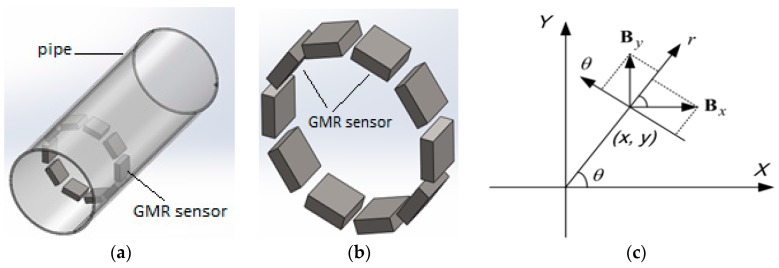
Array of GMR sensor: 3-D design array of GMR sensor (**a**) Overall Probe (**b**) 3-D design of array GMR sensor inside the pipe (**c**) Coordinate transform from Cartesian to Cylindrical coordinate.

GMR array sensors with axial sensitive axes can be located in the center plane of excitation windings to sense the axial components of the magnetic field. With advances in nanotechnology, it is possible to integrate a big number of small GMR sensor elements on the circumference, and then the spatial resolution of detection can be fairly high. A model for an array of GMR sensor is shown in Figure 10.

**Figure 11:**
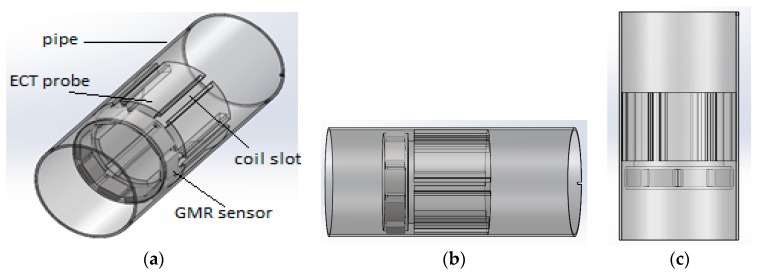
Array GMR sensor located at the ECT probe for pipe inspection (**a**) overall probe (**b**) side view (**c**) top view.

In finite element simulation, the measurements of GMR array sensor can be the axial, radial and azimuth mechanisms of the magnetic flux density. The radial and azimuth components *Br* and *B_θ_* are computed as shown in Equations (11) and (12), whereas, the axial component is the same as *B_z_* in cylindrical coordinates:

(10) $$\theta =\tan^{-1}\frac{y}{x}$$

(11) $$B_{r}=B_{x}\cos\theta +B_{y}\sin\theta$$

(12) $$B_{\theta }=-B_{x}\sin\theta +B_{y}\cos\theta$$

where, (*x*, *y*) are the coordinates of the observation location. The most important magnetic field is the axial component since it also provides a good indication of the circumferential location of the defect in the pipe wall. Figure 11 shows a probe design with an array of 10 GMR sensors for 70 mm pipe inspection.

## 4. Optimization of DSCET Probe

Optimization was applied by using the desirability profile and its functions in the RSM. The highest design parameters with high desirability were chosen as a final design for the ECT probe. The target was to maximize the number of defect detections for axial and circumference defects in pipe defect inspections and minimize the number of GMR sensors while the thickness of excitation coil and probe diameter were set in within a certain range of upper and lower limits for satisfactory results. A solution with high desirability is preferred. Table 1 shows the target value and the upper value for all the parameter responses.

## 5. Results

Experimental tests were conducted to evaluate the system’s functionalities, performance and accuracy of the system in inspection of defects in 70 mm carbon steel pipe. Statistical analysis of the optimization of the DSECT probe design and the interpretation of defect signals are presented and discussed in the following sections.

### 5.1. Analysis of Response Surface Methodology Models

In the probe design for DSECT system, the effects of the probe design parameters including the number of GMR sensor, excitation coil thickness and probe diameter were investigated based on the responses of the axial and circumference defect detection in the pipe defect inspection. The experimental responses of 20 runs in the design matrix along with their corresponding points on the fitted models are based on the RSM are illustrated in Table 2.

ANOVA analyses were executed to validate the accuracy of the empirical models in predicting the defect in pipe inspection using this DSECT system. An *F* ratio for the models of less than 0.05 ensures the empirical model is reflective of the system and appropriate for prediction of the system response, while the value *F* lack of fit for this model was ascertained to be greater than 0.1 to ensure the design of the model is accurate. To ensure accuracy in predicting defects in the pipe, regression analysis and normality tests were done to confirm the accuracy of this model in predicting pipe inspection defects based on different ECT probe parameter designs. After successfully performing all the above tests and confirming the accuracy of this model, a mathematical equation is attained to model the relationship between rate of defect detection in pipe inspections and the ECT probe design parameters.

#### 5.1.1. Axial Defect

Table 3 shows the response of the axial defect model investigated using the analysis of variance (ANOVA) method. The fit of the model was also articulated as the coefficient of determination (*R*^2^) which was observed to be 97.83% of the variability. The “Pred R-Squared” of 0.7776 is in acceptable agreement with the “Adj R-Squared” of 0.9018 in which the difference is less than 0.2. “Adeq Precision” measures the signal to noise ratio. The Model *F*-value of 20.38 suggests the model significance. The *p*-value serves as a tool for inspecting the significance of each coefficient. The values of ‘‘Prob > *F*” are less than 0.05, indicating that the model terms are significant. In this case the ECT probe design parameter *A*, *B*, *C*, *A*^2^, *B*^2^, *C*^2^ are significant factors.

**Figure 12:**
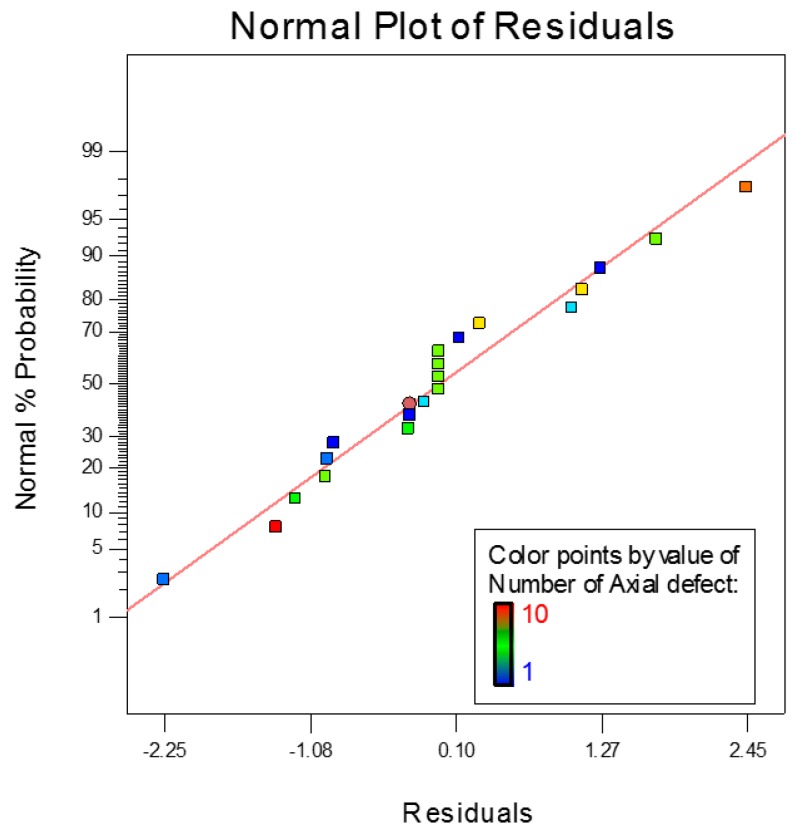
Normal probability plot for axial defect detection.

**Figure 13:**
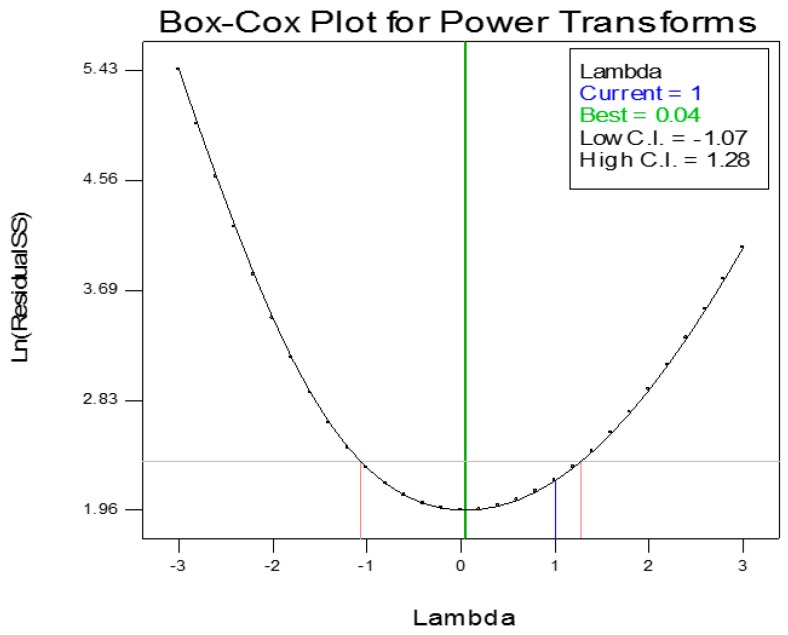
Axial defect detection Box-Cox Plot for power transforms.

Figure 12 shows the normal probability plot for axial defect detection. It is noted that the values of the residuals are very small and closely fitted to the mean line shown in the graph. Therefore, the quadratic model is adequate for modeling the axial defect coefficients. Otherwise, the model should be power altered to other higher level polynomials for accurate results. Figure 13 shows the Box-Cox powers transformation for the axial defect model design. The axial defect RSM model is given by Equation (13) as their actual values:

(13) $$f\left(axial\right)=7.03+2.25A+0.86B+1.79C+0.62AB+0.88AC+0.12BC-1.06A^{2}\;-0.88B^{2}-0.88C^{2}\;$$

**Figure 14:**
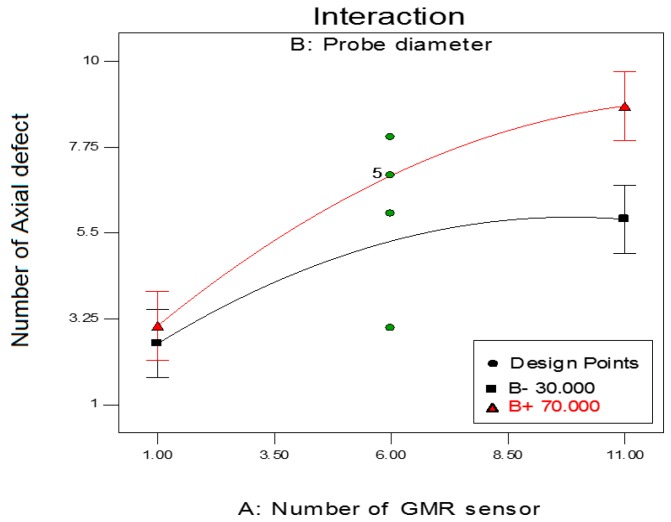
Interaction of probe design factors between probe diameter and the number of GMR sensor on axial defect detection (coil thickness = 12.00 mm).

The interaction relationship parameter between the ECT probe size and the number of GMR sensors in the sensor array to the effect of detection axial defects during the pipe inspection is shown in Figure 14. From the graph, implementation of a maximum number of GMR sensor in the array sensor increases the number of axial defects detected by the ECT probe. The increase of defect detection is significant until the number of GMR sensors in the array of the sensor is 6. For an ECT probe with 30 mm diameter, the number of axial defects detected by the probe is not changed when the number of GMR sensors in array sensor is more than 6. The number of axial defects detected by the DSCET system decreases when the number of GMR sensors in the sensor array is more than 10. For the probe of 70 mm in diameter, the number of GMR sensors in the array sensor is between 1 to 6 sensors, which shows that the axial defects detected by the DSCET system increase almost linearly. However, this increase starts to reduce when the number of GMR sensors used in the array sensor is more than 10.

**Figure 15:**
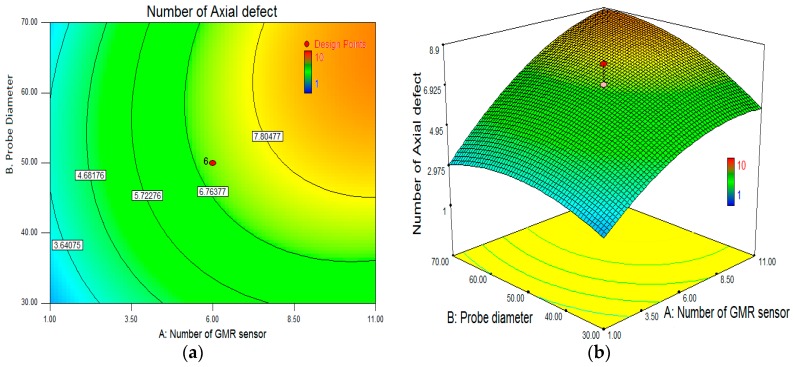
Influence of number GMR sensor and ECT probe diameter in axial defect detection. (**a**) Contour plot; (**b**) 3D surface plot.

The effect of the diameter probe size and the number of GMR sensors in the array sensor, in relation to the effectiveness detection for the axial defect in an inspection of the carbon steel pipe is clearly shown in the 3-D surface and contour plot in Figure 15. With a coil thickness of 12 mm, the contour graph in Figure 15a statistically proposes the probe design diameter and number of GMR sensors used should be 53 mm and six GMR sensors, respectively. The probe diameter size is less than 45 mm which drastically reduces the detection of the axial defects detected by the DSCET system. This is because by decreasing the size diameter, the probe increases the lift-off distance on each GMR sensor in the array sensor. The cylindrical shape of the probe design led to an effective area detected by the GMR sensor. This is influenced by the edge angle of the cylinder, thus to maximize the defect detection in the pipe is optimal with regard to the GMR sensor that needs to identified. ANOVA analysis in Table 3 also shows the size of the probe diameter and the number of GMR sensors has a significant effect in determining the effectiveness of the DSCET system for axial defects in the attempt to detect defects during the carbon steel pipe inspection.

#### 5.1.2. Circumference Defects

Table 4 shows the ANOVA analysis of circumferential defect detection for carbon steel pipe inspection. It summarizes the influences of each probe design parameter and their interactions fitted as a second-order quadratic model for circumference defect inspection in pipe inspection. The model is formed for 97.73% confidence level with an appropriate model reduction. The model *F* value for the circumference defect is 19.97. The *p* value of 0.0001 implies that the model is significant with negligible influence of noise.

On careful examination of the *F* and *P* values, it can be observed that the probe design parameters *A* (number of GMR sensors) has the most significant effect on circumference defect detection followed by parameters *B* (probe diameter) and *C* (coil thickness). The *F* test also projects the relevance of a certain number of GMR sensors in the array sensor for both types of defect detection.

The statistical accuracy is also evaluated to determine that the predictive coefficient of circumference defects is 0.9773. Adequate precision, which is a measure of the S/N ratio, is found to be 14.463, which confirms the accuracy of the model. Equation (14) is the empirical equation for the prediction of circumference defect detection as a function of the independent probe design parameter variables which are the number of GMR sensors, probe diameter and the thickness of the probe coil:

(14) $$\begin{array}{l} f\left(circumference\right)\\ \;=8.02+2.30A+0.79B+1.99C+0.5AB+0.5AC-0.25BC\\ \;-1.19A^{2}-0.83B^{2}-1.01C^{2} \end{array}$$

**Figure 16:**
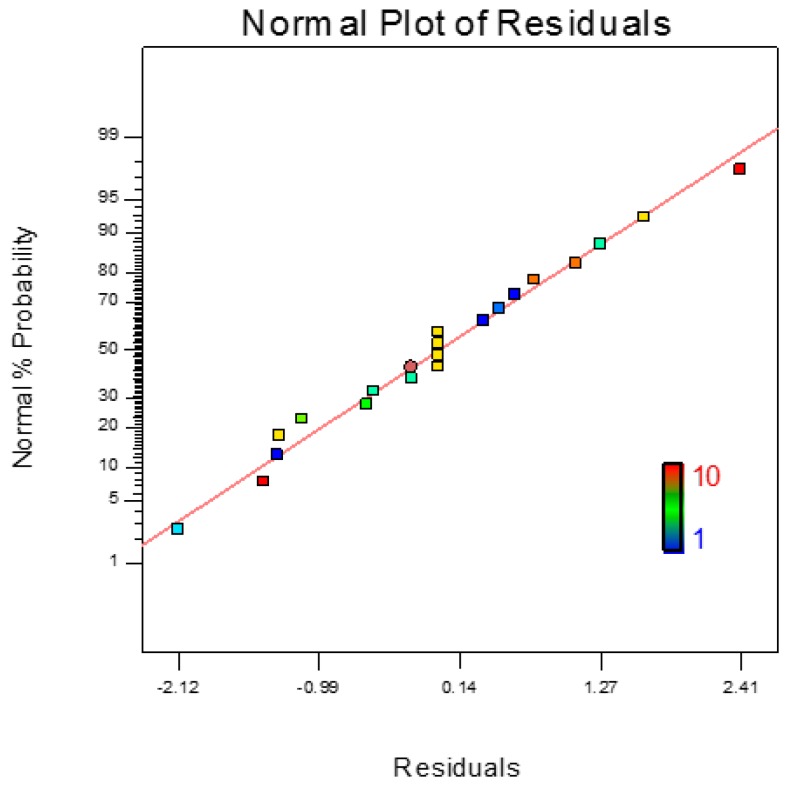
Normal probability plot for circumferential defect detection.

**Figure 17:**
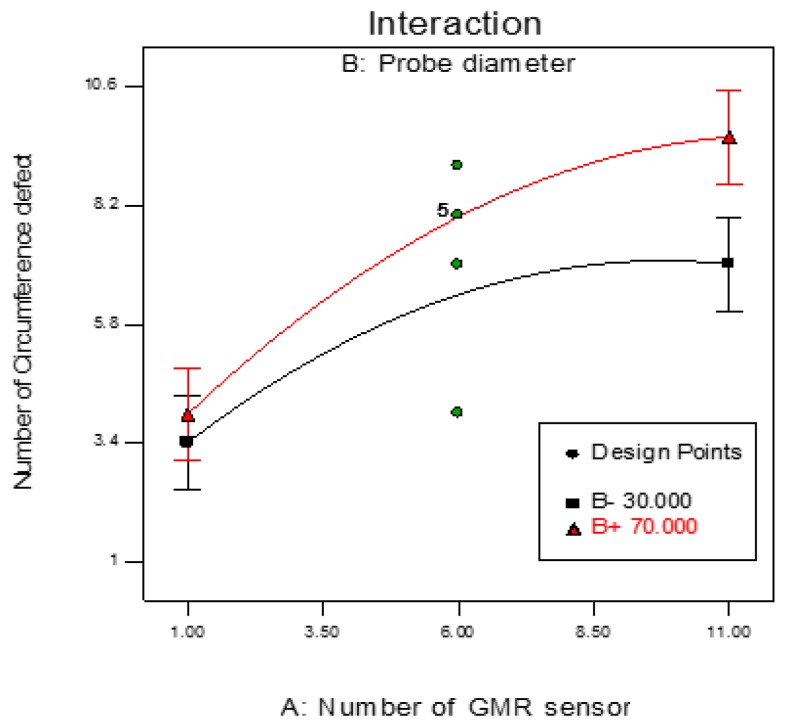
Interaction of probe design factors between probe diameter and the number of GMR sensor on circumference defect detection (coil thickness = 31.00 mm).

It can be seen that interactions between the probe parameter designs affect the variance of the circumference defect detection. Figure 16 shows the normal probability of circumference defect detection lies close to the mean line which evidences that the values of the residuals are marginal. Figure 17 shows the interaction between the factor involving the number of GMR sensors and probe diameter and the detection of the circumference defects. Therefore, all the parameters of the probe design are considered when optimizing the detection rate of the circumference defect.

**Figure 18:**
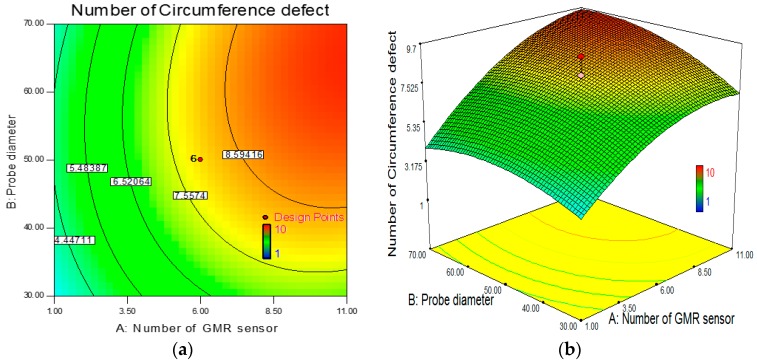
Influence of number GMR sensor and ECT probe diameter in circumferential defect detection. (**a**) Contour plot; (**b**) 3D surface plot.

The contour and 3-D plots in Figure 18 are obtained using the fitted model by ensuring the least effective independent variable is at a constant value while changing the other two variables. In this study, a Box-Behnken design with three levels of variables was applied.

### 5.2. Optimized Probe Design

A desirability analysis is conducted to discover the optimum conditions parameter design by taking into account all responses. Using the Design Expert software, a numerical optimization was performed to explore the design space by utilizing mathematical models to determine the factor settings that fulfill the defined goals. The highest desirability value is 0.71, which suggests that the maximum axial and circumference defect detection can be obtained when the ECT probe design parameters are a six GMR sensor used in the array sensor, the probe diameter is 58.45 mm and the coil thickness is 22.34 mm.

**Figure 19:**
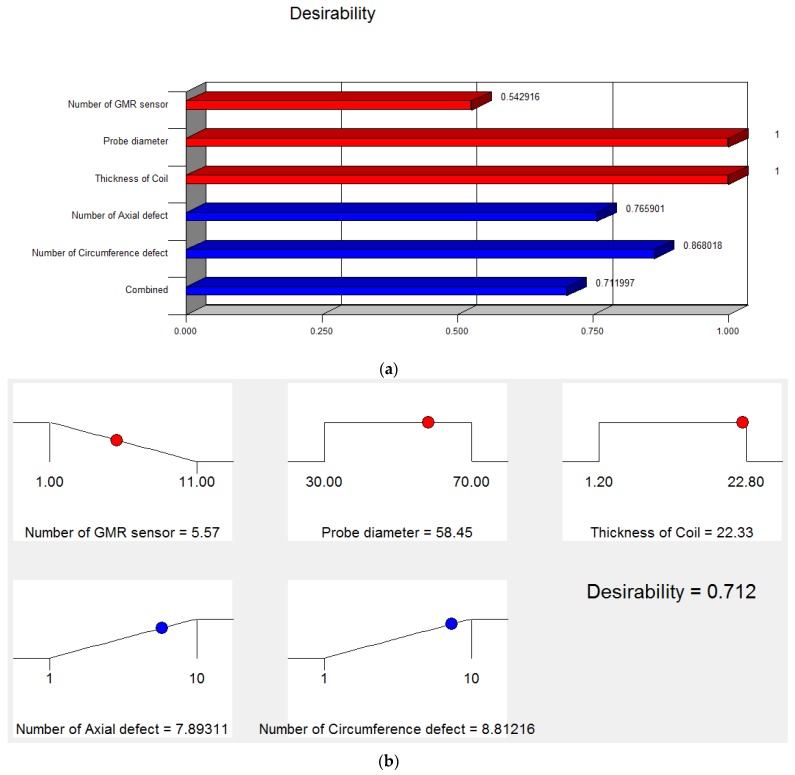
Optimization solution for ECT probe design (**a**) Desirability graph (**b**) Ramp function graph.

**Figure 20:**
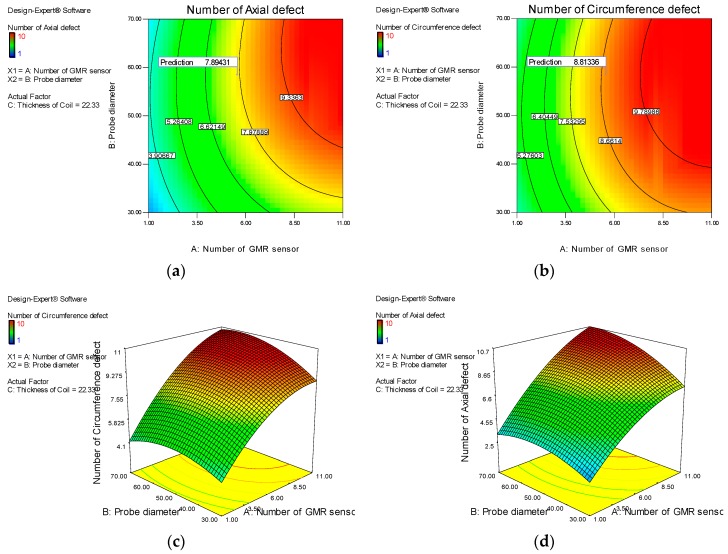
Prediction of defect detection under optimum ECT probe design. (**a**,**b**) shows the contour graph for axial and circumference defect detection; (**c**,**d**) show the and 3-D graph for the axial and circumference defect detection.

**Figure 21:**
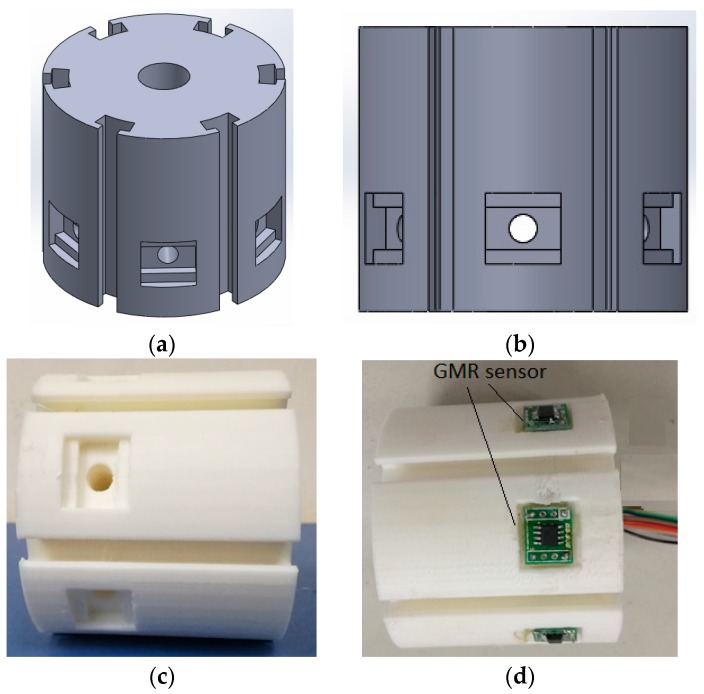
ECT probe design for DSECT system based on optimum parameter design (**a**) Trimetric view (**b**) Left view (**c**) Probe prototype (**d**) Probe with GMR sensor.

Figure 19 shows the desirability and ramp function graph for the factor and the response in the optimization of ECT probe design while Figure 20 shows the contour and 3-D graph for the axial and circumference defect detection with the optimum ECT probe design parameters. The final design for the ECT probe is based on the optimum factor design shown in Figure 21. Since the optimum number of GMR sensors is six, the modification of the sensor configuration is made where the GMR array sensor is located in the center of the probe coil. The new configuration makes the design more compact with the same effectiveness for the 70 mm pipe inspection since the parameter design still uses the optimum probe design parameters.

### 5.3. Simulation of the Axial and Circumference Defect for Carbon Steel Pipe

A finite element model is developed to predict the response signals of the probe designed for the inspection of carbon steel pipe with axial and circumference defects. Both types of defect are modeled, and the corresponding signals are compared. GMR sensor array elements are used for the pickup sensors. The inner diameter of the simulated pipe is 70 mm, and the wall thickness is 5 mm. The pipe material is carbon steel, and its conductivity is 6.99 × 10^6^ S/m. The dimensions of the defects are shown in Table 5.

The optimized design probe parameters used in this inspection are a number of GMR sensors of six in the array sensor, a probe diameter of 60 mm and a 22.34 mm excitation coil, respectively. The ECT probe is excited by a current source of 3 × 10^6^ A/m^2^ with 60 kHz frequency. The probe moves axially within the carbon steel pipe at a speed of 0.8 m/s. The GMR sensors located around the excitation coils will detect the magnetic flux density induced by the rotating fields.

**Figure 22:**
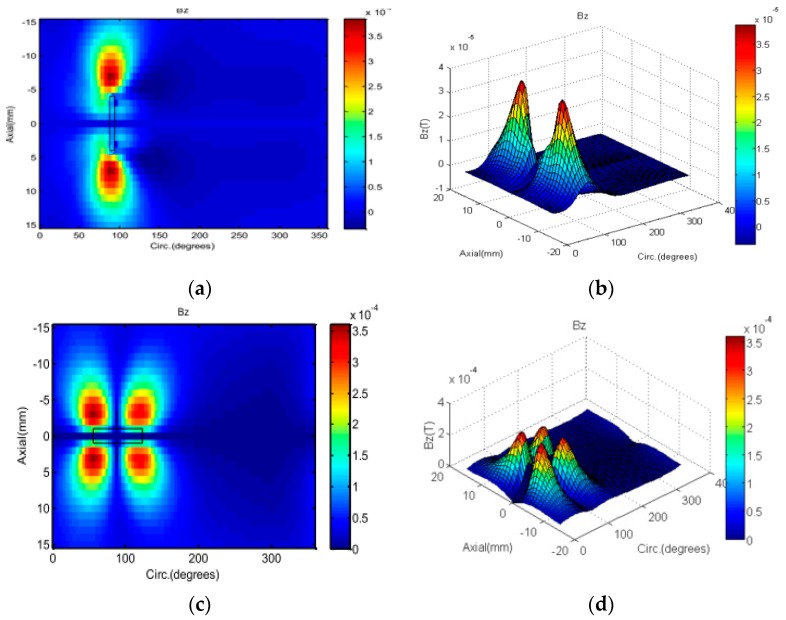
Axial magnetic flux density due to different defect with 100% depth measure by GMR sensors: axial defect (**a**) 2D; (**b**) 3D, circumferential defect (**c**) 2D; (**d**) 3D.

The axial components of the magnetic field, due to the axial defect (12 mm × 2 mm) and circumferential defect (2 mm × 12 mm) 100% depth measured by an array of GMR sensors are shown in Figure 22. Since the main source field has a rotating radial magnetic field, the axial component of the magnetic field is generated only in the presence of a defect. At the defect-free position, the induced eddy currents generates a radial field opposed to the source field. When a defect is present, the eddy current is disturbed and an axial magnetic field component appears due to the eddy current re-distribution. From the 2D images, the profiles of both defects are easily determined and classified.

### 5.4. Experimental Results for Axial and Circumference Defects

A carbon steel pipe with a diameter of 70 mm was inspected using the DSECT system and the prototype probe. The probe design is based on the optimized parameters obtained from the Design Expert optimization where the ECT probe design parameters are six GMR sensors used in the sensor array, a probe diameter of 58.45 mm and a coil thickness of 22:34 mm. Artificial axial and circumference defects are fabricated on the carbon steel pipe using Computer Numerical Control (CNC). The size dimensions of the axial defect are 2.0 mm width and 13.7 mm high, while the size of the circumference defect is 13.7 mm width and 2.0 mm high.

**Figure 23:**
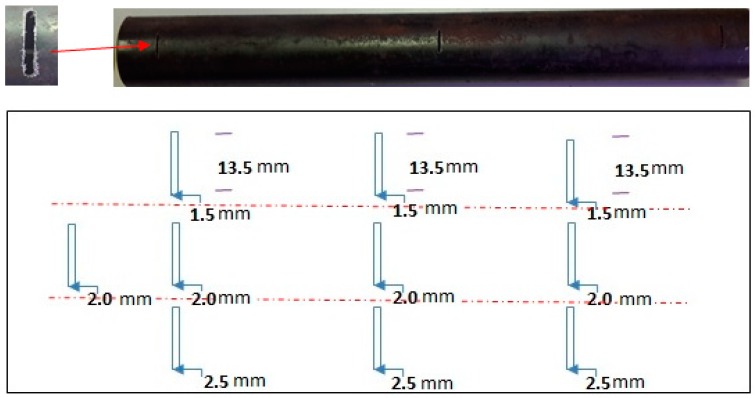
Geometric dimensions of the circumference defects on the carbon steel pipe.

**Figure 24:**
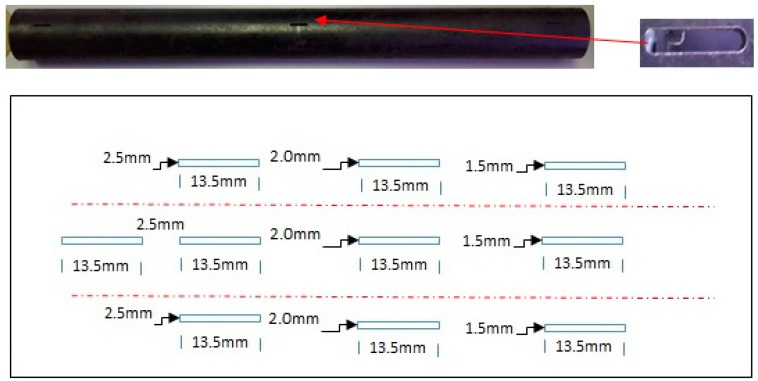
Geometric dimensions of the axial defects on the carbon steel pipe.

Two sets of carbon steel pipe samples with 10 axial and circumference defects, respectively, were used to evaluate the efficiency of the DSECT and the probe design. Figure 23 and Figure 24 show the carbon steel pipe samples and the layout of the axial and circumference defect positions.

**Figure 25:**
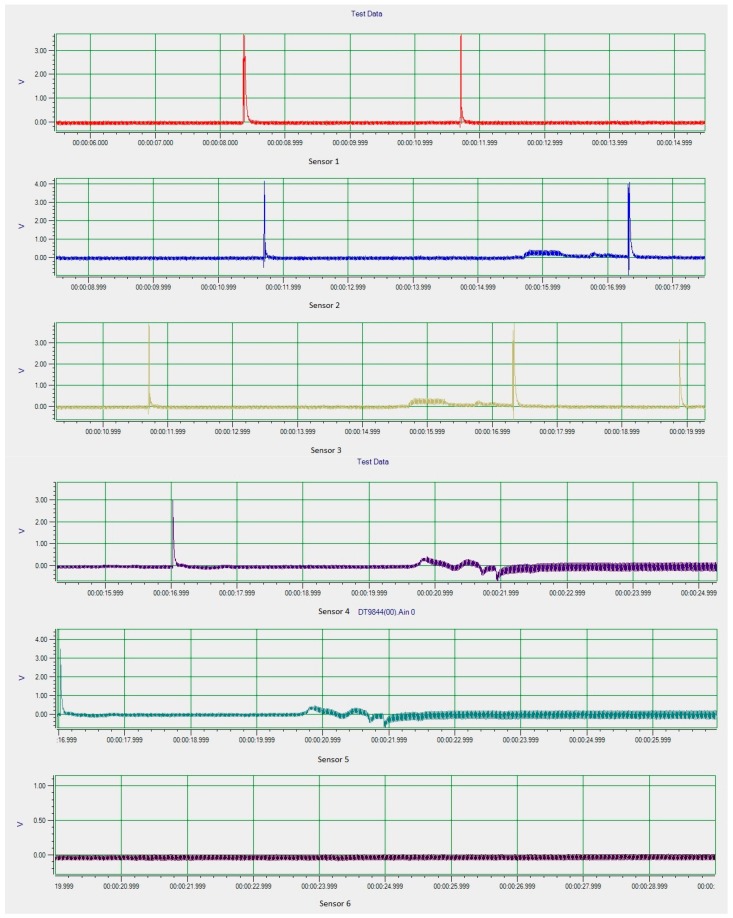
Experimental results for circumference defects.

**Figure 26:**
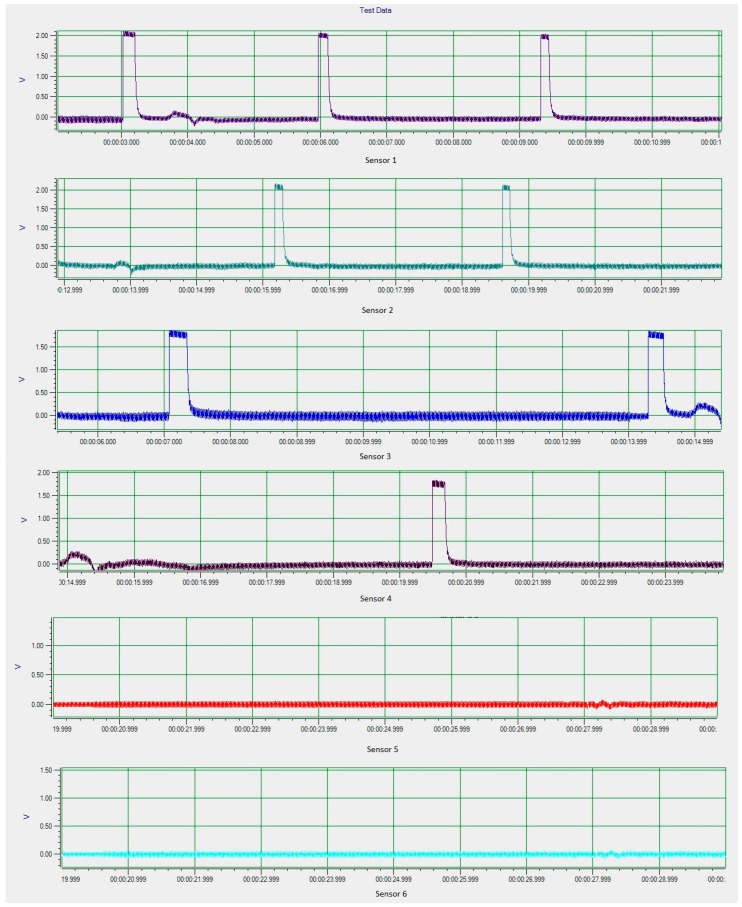
Experimental result for axial defects.

Carbon steel pipe with 100% of the tube wall for both axial and circumference defects are test using the DSECT system and the proposed ECT probe prototype. The frequency of the current excitation for generating a rotating magnetic field is 50 kHz. The output from the array of GMR sensors in the ECT probe for the inspection of the artificial defects on carbon steel pipe is shown in Figure 25 and Figure 26.

From the experimental inspection the results show the DSECT system is able to detect both types of defect. The detection rate for both types of defect is high, at almost 97%. This indicates the proposed system and probe design are highly sensitive for different defect orientations. Detailed analysis on the signals for axial and circumference defects shows that the indicating the width of the signal generated by an axial defect is larger compared to the defect signal generated by a circumference defect. This indicates that different sizes of defect generate different signal profiles, which is useful for defect classification. The experimental results validate the simulation model and demonstrate the feasibility of the probe design.

### 5.5. Comparison Accuracy of the Probe Design

A confirmation test was conducted using the optimum probe design conditions for the axial and circumference defect detection. The results are compared with the predicted values from the mathematical model and tabulated in Table 6. These are the results of the optimum ECT probe design conditions considering all the responses. The results show a close agreement between the predicted and experimental inspection results.

## 6. Conclusions

This paper recommends a distributed system for pipe defect inspection using the non-destructive eddy current testing method. The DSECT system architecture allows the connection of up to 32 sensors which are regarded adequate in terms of the optimum number of GMR sensors in a sensor array and six for the inspection of three inch carbon steel pipe. The implementation of the optimized design using the proposed system significantly increases the measurement accuracy and scope of the inspections. The optimization analysis indicated that the combination of a minimum of six GMR sensors in an array sensor, a diameter probe of 58.45 mm and an excitation coil thickness of 22.34 mm is the best combination for maximum defect detection in a 70 mm carbon steel inspection. In further works, an analytical analysis and the ability to compensate unwanted signals using an intelligent algorithm are necessary for a comprehensive defect profile analysis method within pipe structures. The limitations of the size of the defects that can be detected by the system must be investigated in order to ensure the reliability of the system for pipe inspection.

## Figures and Tables

**Table 1:** Target value and limit for optimization of DSCET probe design.

Probe Design Parameter and Respond	Target	Lower Limit	Upper Limit
Number of GMR sensor	Minimize	1	11
Probe diameter (mm)	Minimize	30	70
Coil thickness (mm)	Minimize	1.2	22.8
Axial defect	Maximize	1	10
Circumference defect	Maximize	1	10

**Table 2:** Experimental design and results (uncoded factors).

Run	Factor 1 A: Number GMR Sensor	Factor 2 B: Probe Diameter (mm)	Factor 3 C: Thickness of Coil (mm)	Response 1 Axial Defect Detect	Response 2 Circumference Defect Detect
1	6.00	50.00	6.16	1	1
2	6.00	50.00	12.00	7	8
3	11.00	30.00	1.20	3	4
4	6.00	50.00	12.00	7	8
5	1.00	30.00	1.20	1	1
6	1.00	70.00	1.20	1	2
7	11.00	30.00	22.80	7	8
8	6.00	16.36	12.00	3	4
9	2.41	50.00	12.00	1	1
10	11.00	70.00	1.20	5	6
11	6.00	50.00	30.16	9	10
12	1.00	70.00	22.80	2	3
13	1.00	30.00	22.80	2	4
14	6.00	83.64	12.00	7	8
15	6.00	50.00	12.00	6	7
16	6.00	50.00	12.00	8	9
17	6.00	50.00	12.00	7	8
18	6.00	50.00	12.00	7	8
19	14.41	50.00	12.00	8	9
20	11.00	70.00	22.80	10	10

**Table 3:** ANOVA table for axial defect detection response surface quadratic model.

Source	Sum of Squares	df	Mean Square	*F* Value	*p*-Value Prob> *F* Prob > *F*	Remarks
Model	164.81	9	18.31	20.38	<0.0001	significant
*A*-Nu sensor	69.34	1	69.34	77.16	<0.0001	significant
*B*-diameter	10.07	1	10.07	11.21	0.0074	significant
*C*-coil thickness	43.79	1	43.79	48.73	<0.0001	significant
*AC*	6.13	1	6.13	6.82	0.0260	significant
*A*^2^	16.14	1	16.14	17.96	0.0017	significant
*B*^2^	11.20	1	11.20	12.46	0.0054	significant
*C*^2^	11.20	1	11.20	12.46	0.0054	significant
Residual	8.99	10	0.90			
Lack of Fit	6.99	5	1.40	3.49	0.0980	not significant
Pure Error	2.00	5	0.40			
Cor Total	173.80	19				

**Table 4:** ANOVA for the circumference defect detection response surface quadratic model.

Source	Sum of Squares	df	Mean Square	*F* Value	*p*-Value Prob > *F* Prob > *F*	Remarks
Model	177.10	9	19.68	19.97	<0.0001	significant
*A*-Nu sensor	72.45	1	72.45	73.53	<0.0001	significant
*B*-diameter	8.43	1	8.43	8.55	0.0152	significant
*C*-coil thickness	53.92	1	53.92	54.73	<0.0001	significant
*A*^2^	20.30	1	20.30	20.61	0.0011	significant
*B*^2^	10.01	1	10.01	10.16	0.0097	significant
*C*^2^	14.71	1	14.71	14.93	0.0031	significant
Residual	9.85	10	0.99			significant
Lack of Fit	7.85	5	1.57	3.93	0.0798	not significant significant
Pure Error	2.00	5	0.40			
Cor Total	186.95	19				

**Table 5:** Simulation defect parameters.

Type of Defect	Dimension		
	Width (mm)	Length (mm)	Depth (mm)
Axial	12	2	5
Circumference	2	12	5

**Table 6:** Comparison of the predicted and experimental results.

Defect	Prediction	Experimental	Error (%)
Axial	7.89535	8	−1.32
Circumference	8.8141	9	−2.11
